# Spirit of the British and American Periodicals

**Published:** 1840-04-01

**Authors:** 


### Spirit of the British and American Periodicals, &c.

### Pressure in Cases of Chronic Hydrocephalus.*

Mr. Barnard, surgeon to the Walcot Dispensary, doubts whether hydrocephalus is ever idiopathic. He seems to be of opinion that there is inflammatory action in the first instance, the hydropic effusion being an effect of it. Be that as it may, when the head begins to enlarge, an opposite condition, one of weakness and relaxation, has commenced, and a different method of treatment becomes necessary.

Mr. Barnard observes:—"The first idea which led me to adopt the peculiar plan of treatment which shall be detailed, was from observing some adults with heads of such a size as could have been caused by no other than hydrocephalus in infancy.

It occurred to me at once that, although we possessed no cure for the disease, nature, amidst her curious arcana, did; and upon enquiring minutely into the history of some of these cases—and one particularly in the progress of cure—I found that it was nothing more nor less than a comparatively early union of the bones of the head, thereby forming a natural and most efficient bandage, commencing the process of compression, which I, by artificial means, propose." The principle on which he acts in these cases is, to render that support to the parietes of the head which seems to be altogether wanting, and which nature evidently points out as the only remedial measure with any chance of success.

The following is one of six cases related by our author in illustration of his views. They would all appear to have been equally fortunate in their results.

*Case.*—"A child, twelve months old, whose head was observed to have been increasing in size for the previous four months, now measured in circumference nineteen inches; the fontanelles and sutures were much open, but the parietes were not particularly distended, although fluid could be distinctly felt; the whole head was loose and flabby, and the bones as it were floating; the countenance shrunk and pallid, and the body generally much emaciated; pupils dilated and one eye-lid dropped, which the patient seemed perfectly unable to lift; slight convulsions occasionally; but usually it lay in a quiet, sleepy state; took its food rather voraciously; the bowels irregular, sometimes loose and sometimes costive, but the excretions always unhealthy. It had been attended by three separate medical gentlemen, who had given up the case as hopeless.

The head was shaved, and the adhesive bandage applied on the 10th of March; and, although compression was carried to a much greater extent than I have ventured on in any other case, no untoward symptom followed.

For three months no particular change in the state of the child took place; at this time, June the 10th, although the plasters were still firm, I thought it well, from the growth of the hair and other circumstances, to renew them, which accordingly I did. The bowels were still irregular, requiring occasionally a dose of castor oil, which answered the purpose exceedingly well, and no other medicine was given; the appetite still continued good; the food consisted of bread and milk and arrow-root.

July 15th—The plasters again renewed; no particular change in the state of

---

* Cases of Chronic Hydrocephalus in the Head, &c. By J. F. Barnard, M.R.C.S., &c.

the patient. The head appeared rather firmer, but no alteration in size; the bowels more regular, and evacuations somewhat improved. Diet ordered to be altered to beef-tea and jellies.

Sept. 5th—Both eyelids in perfect action; no convulsions; bowels regular, and evacuations healthy; countenance expressive of ease and comfort; can sit up with little assistance, and appears lively; head much firmer, and reduced in size half an inch; gains flesh.

Oct. 29th—The head is firm and the sutures quite closed; the child begins to walk about, as yet rather staggering. Plasters left off. The child continued to progress, and in three months was restored to perfect health."

The pamphlet and the plan deserve attention.

---

### ON RETROVERSION OF THE UTERUS, AND A NEW METHOD OF TREATING THAT AFFECTION. BY CHARLES HALPIN, ESQ.*

Mr. Halpin passes in review the modes of treating retroversio uteri that have been recommended or practised, for the sake of introducing a case and a new method.

We need not detail the particulars of the case, which was a decided one of retroversion of the uterus, about the fourth month of pregnancy. Mr. Halpin first tried two fingers of the right hand in the vagina—then two fingers of the right hand in the vagina, and the thumb in the rectum. The patient exclaimed that "he was bursting something in her inside," and he was obliged to desist.

"I now relinquished all hope of being able to return the uterus safely by any introduction of the fingers or hand into the vagina or rectum, and retired with Dr. Finlay to consider our difficulties. I communicated my impressions to him; which were, that on bringing my fingers to bear upon the tumour, I found I could always move it to a certain extent, but after it reached this it became fixed, and I could feel the parietes of the uterus yielding under the very small point of contact afforded by my fingers; this gave rise to the sensation of rupturing the parts complained of.

I now saw clearly that my only chance of rescuing this woman from her perilous state would be, in the use of some instrument which could be brought to bear equally on all parts of the tumour, and with which sufficient power could be applied to raise it fairly above the promontory of the sacrum.

It instantly occurred to me, that *with the assistance of a bladder I should be able to inflate the pelvis, and thus raise its contents into the abdomen.* We acted on this suggestion. I attached a small recent bladder to the tube of a stomach-pump, with an air-tight piston, and having immersed it a few moments in warm water, to bring it to the heat of the body, I introduced it, empty, into the vagina, between the fundus of the uterus and the rectum. Retaining it within the vagina by holding my hands firmly across its orifice, Dr. F. inflated it slowly and steadily. After a time she complained of a sense of tension or bursting, but no pain. We then ceased throwing air into the bladder, allowing what was in already to remain, keeping up, as it did, a steady, equal, well-directed pressure on the tumour. After the expiration of five minutes we threw more air into the bladder, when the patient exclaimed slowly, 'Oh, now you are forcing something up to my stomach!' I retained the bladder some time longer in its situation; and then, previous to withdrawing it, permitting the escape of some air, I introduced my finger, and had the satisfaction of finding that the tumour was no longer in the pelvis, and that the os uteri lay within reach of my finger,

---

* Dublin Journal, March, 1840.

pointing downwards and backwards. I then, and not till then, removed the apparatus."

In his remarks, Mr. Halpin states that he considers this instrument applicable to the retention of the uterus in its normal position, when replaced. The retroversion having been rectified, he would introduce, as a pessary, a gum-elastic bag constructed on this principle, and inflate it to a proper state of distention. It will remain without producing the least annoyance to the patient; and cannot, from its nature, obstruct the free passage of either urine or fæces; whilst it will render the descent of the uterus within the pelvis a matter of impossibility.

---

### Some Remarks on Aneurism of the Internal Carotid Artery. By William Henry Porter, Esq.*

The object of Mr. Porter, in a long and valuable paper, is to shew the unfavourable position of the internal carotid artery, when affected with aneurysm—unfavourable, we mean, for an operation. After alluding to the occurrence of want of coagulation and to suppuration in the sac, he remarks:—"In considering the causes that may interfere with, or prevent the successful issue of an operation, and the recovery of a patient, it is rather an unusual view of the subject to regard the situation of the tumor as exercising a very decided influence, or to aver that the principle on which we treat an aneurysm in the ham or in the thigh with almost a certainty of success, may not be applicable to the management of a similar affection in a different locality. Yet such, I fear, is practically correct; and although other collateral circumstances must be taken into account, as contributing their share in the production of this unpleasant result, yet I believe the internal carotid artery will be found so placed, as to afford a peculiar facility for the occurrence of these untoward complications, and that operations performed for the cure of aneurisms of this vessel, are attended with a degree of uncertainty scarcely to be expected elsewhere."

Mr. Porter points out the main circumstance that favours coagulation in the sac, viz. the blood getting into it sluggishly, and without impulse through the anastomotic branches. The fluid in the sac is therefore pretty quiescent, its natural tendency is to become coagulated, and the coagulum thus formed, being restrained by the sac and its coverings from extending itself in any other direction, is pushed towards the point where there is least resistance; that is, against the open vessel which it compresses and causes to become obliterated, just as would happen to any bleeding artery under the continued pressure of a clot. From this, and from other considerations, Mr. Porter thinks it will be conceded that any thing capable of disturbing the blood within the sac, will interfere with the process of coagulation, and thereby delay, if it does not prevent, the cure; and that any condition or position of the sac, which will allow the pressure of its contents to be directed otherwise than against the injured vessel, must have a similar effect. Either of these circumstances taken singly, may prevent the cure; when both are combined, success is scarcely to be hoped for.

"When pulsation," he adds, "is observed to return in the aneurismal tumor in a short time after the application of the ligature, it is obvious that the great object of completely cutting off the impulse of the heart has not been attained; that blood, to a greater or less quantity, not only enters the sac, but that some is returned again from it, and that the blood within it is constantly disturbed, and therefore kept in a state unfavourable to coagulation. This phenomenon of

* Dublin Journal, March, 1840.

the re-appearance of pulsation, has of late years been frequently observed, and many of the causes that may conduce to it are now well known; some of them being of a nature entirely to prevent the cure of the aneurism, such as the existence of two large trunks in the limb, by an irregular arterial distribution, or where one or more large branches arise from, or otherwise communicate with the sac, and others which only delay the sanative process, but do not (at least necessarily) completely interfere with it. These latter are, firstly, the circumstance of the ligature being tied so loosely as not only not to stop the current of blood through the vessel, but not completely to cut off the impulse of the heart from the distal circulation; and what is of more importance, as bearing on the present subject, the existence of such an extensive and free communication by anastomosis as will convey to the tumor, by a circuitous route, the impulse which the ligature was intended to remove. I know not how far such a communication may be established in an aneurismal limb by a preternatural increase of size in the collateral vessels: such has been spoken of by authors, but I have no evidence of its existence in any one particular case to an extent sufficient to produce the phenomenon alluded to. However, I believe this unhappy condition obtains, with respect to aneurisms of the internal carotid artery in the neck, and that the free and extensive anastomoses through the vessels of the brain in their natural and normal state, will be quite sufficient, in some instances, as in the following case, to delay the process of cure, in others perhaps, to prevent it."

This may be looked on as the pith of Mr. Porter's paper, and it is to establish this that he introduces several cases, all interesting, some more and some less conclusive. These cases, tend, he thinks, and we agree with him, to shew that aneurysms of the internal carotid are unfavourably placed for the accomplishment of one part of the cure; that a ligature on the trunk does not entirely cut off the impulse of the heart from the diseased vessel; that such impulse may be conveyed through the circulation of the brain in its normal state; and that the effect of this impulse must be, to disturb the blood within the sac, and delay, if it does not prevent, its coagulation.

But Mr. Porter points out another circumstance in connexion with aneurysms of the internal carotid, which is far from unimportant.

"In a limb, when the aneurismal sac becomes distended with blood after operation, the pressure exercised thereby must be directed against the injured vessel; the structures external to, and surrounding such sac are, many of them, inelastic, all more or less resisting; they will not permit a growth or extension of the tumour in any direction towards them, and consequently when the sac is filled, and more particularly when its contents are solidified, it must not only press against the ruptured vessel, but compress it to an extent and degree to occasion its obliteration. But with respect to the internal carotid, the case is widely different. However covered with fascia, and muscle, and skin, and other resisting structures externally, it is wholly unprotected in the direction of the pharynx, for, on making a vertical section of the head and neck, and dissecting from within outwards, I find that the internal carotid is very close to the pharynx. In its passage upwards, from the bifurcation to its entrance into the skull, it obliques slightly backwards and inwards, having behind it the sympathetic nerve and first cervical ganglion, where it rests against the spine: external it has the styloid process, the three styloid muscles, the digastric, the mastoideus, and the different layers of fascia; a little in front it has the stylo-pharyngeus muscle, but internally, or towards the pharynx, it has nothing but the mucous membrane, the contrictor of the pharynx, some very loose cellular tissue, and the twigs of the superior laryngeal nerve; thus the aneurismal sac has ample room to grow and increase inwardly, and consequently the pressure it is forced to make on the opening in the vessel may be so trifling, as not in any way to lead to its obliteration. This circumstance may explain (although in Mr. Green's case it does not appear that the mouth and pharynx were ever examined) why that case proved to be what Scarpa might term an imperfect cure, and why in the case of Markay, in five weeks after the vessel was tied, and the direct force of the heart cut off, there should have been no advance whatever in the process by which the artery might be obliterated."

*Defective Tendency of the Blood to Coagulate.*

Mr. Porter asks :—" Is these a pathological difference in the blood of different individuals, giving to that of one a greater or less tendency or disposition to coagulate, than to that of another ? If there is such a difference of condition or constitution, a knowledge of the fact, and more particularly of the causes or circumstances that lead to its production, might prove of incalculable benefit in the management of disease in general, but more particularly those of the circulating system. And that there is such a difference, I am strongly disposed to believe, although being totally unprepared with satisfactory proofs, I dare not offer it even as an hypothesis—but rather as a suggestion that may lead others (as opportunity may offer) to investigate the pathology of the blood as promising to lead to invaluable practical results."

Surely this is no new idea. The cases that have at different times been published of a remarkable tendency to hæmorrhage, more particularly Mr. Blagden's case, have been looked upon as instances of what Mr. Porter mentions. We have indeed, in our own physiological lectures, been accustomed for some years to advert to this very point, and to cite those cases as examples of the imperfect power of coagulation which obtains in some individuals.

---

### Mr. Lee on Nervous Disorders.

In a second edition of Mr. Lee's little work there is an Appendix, containing some interesting cases, of which we shall give a short account in this article.

1. *Paralysis succeeded by incessant Movements.*—This was a Florentine girl, aged 17, menstruating irregularly, but otherwise healthy. She had a fall and injured her neck, where an abscess formed. Paralysis of the left arm and leg succeeded. Subsequently paralysis of the right side occurred, in which state she was brought to the hospital in November. The intellectual, respiratory, and digestive functions remained unimpaired. The case was considered spinal inflammation. Leeches, blisters, low diet, and strychnine were employed. They produced no apparent effect. In consequence of a sudden mental shock, the paralysed members became affected with constant involuntary movements, which lasted several weeks. Mr. Lee suggested to the physicians that the disease was hysteria. The diet and treatment were altered, and she recovered.

2. *Incessant Motion of the Head.*—This and two or three other cases are taken from Bell and Bright. They need not be noticed here.

3. *Complicated Nervous Affection.*—Cæsar Pineiro had enjoyed good health till the age of ten, when he was greatly frightened, soon after which he began to experience the most strange and anomalous symptoms—drowsiness—convulsions of an epileptic character—chorea—loss of sense—jactitations, &c. succeeded each other, or alternated in great irregularity. One constant symptom was uneasiness in the chest, stretching from the left hypochondrium to the mouth. Various medicines were tried with questionable effect. It was curious that, out of hundreds of attacks, only five occurred out of his own house. He was sent from home, put to reside with a priest, and had no more fits. There was tenderness on pressure over one of the dorsal vertebræ.

4. *Intermittent Aphonia.*—Mad. B. aged 46, married. Lives in the country, and takes regular exercise, and menstruates freely. At the age of 30, she suddenly lost her voice, at 12 o'clock, without fever, pain, or local uneasiness. The voice returned at night, but was lost the next day at the same hour. At the end of three weeks the Aphonia Periodica disappeared. In a few years afterwards, she had returns of the aphonia, the attacks coming on daily, and the period lasting for a fortnight or three weeks. It was in 1817, that the attacks took the following character. Every year, in February, the attack was preceded by rigors and yawnings, exactly at mid-day, without any alteration in the pulse, or local pain. The aphonia then becomes almost complete. There is a sense of constriction in the epigastrium, from whence the impediment of speech seems to proceed. The aphonia continues all the evening. She goes to sleep, and awakes quite well. The periodicity continues as regular as the sun. By long repetitions of these attacks, the general health became enfeebled. She took eight grains of quinine every morning without any effect. No other medicines stopped the paroxysms, which generally continue to recur daily for three to seven months every year, after which she regains health till the succeeding February. It was expected that the disease would stop at the turn of life.

We think this periodical aphonia was connected with some cause attached to the locality of the lady's residence, and was of the aguish character. The measures, as may be easily supposed, were anything but energetic in therapeutics.

5. *Surprising Cure by Homœopathy.*—A young girl was admitted into the Hôtel Dieu, on the 4th of January, who had lost her voice completely since the middle of November preceding. The usual remedies produced no effect. She was then placed in the homœopathic department, and two starch pills were given—one in the presence of the physician, and the other four hours afterwards. In a few minutes after the first pill was exhibited, the patient evinced anxiety, pain and uneasiness in the region of the heart, perspiration, with heat of skin, and a florid efflorescence on the surface. The second pill aggravated all these symptoms, with the addition of hiccup. She afterwards fell asleep, and, on awaking, found that she could talk as loud as any of her fellow-patients.

This is a good specimen of the wonderful cures performed by homœopathy, and animal magnetism. If, as Plato believed, the mind is the cause of half our diseases, we see no reason why the same agent may not remove those diseases which it has originated. In truth, much of the etiology and therapeutics of human maladies is under the influence of the imagination. Hence it is that a physician of reputation will cure complaints with the very same remedy that has failed in the hands of less celebrated practitioners. It is wisely ordained that the tyrant death, or their infirmities, permit but a very narrow range and limited existence to talent and reputation. Were it otherwise, one-half of the world would be starved by the overgrown wealth of the other!

---

### A Case of Inflammation of the Schneiderian Membrane, extending along the Olfactory Nerve to the Brain, and producing an Abscess in its Interior. By Henry James Johnson.

The following case appearing to present some features of interest, I have ventured to lay it before the readers of this Journal. I regret that, from circumstances which it is unnecessary to mention, the account of it is so imperfect.

*Case.* I was requested by a lady, in the Spring of last year, to see her maid, a person of fifty years of age or upwards. She complained of discharge from her left nostril. The discharge was yellow in colour, purulent in character, and rather " sickly" to the smell (her own) than decidedly offensive. The left side of the nose was more protuberant than the right, and a very faint erythematous redness was upon the skin. The swelling appeared to be in the soft parts covering the nasal bones, rather than an expansion of those bones themselves. There was some tenderness on pressure. On examining the nostrils, the Schneiderian membrane in the left was redder than natural and tumid, but no ulceration was perceptible, nor could the probe light on any portion of exposed bone. The fauces presented nothing unusual. There was no affection of vision, nor was there any pain in the head, or disturbance of it.

The countenance of the patient was sallow and unhealthy. Her tongue was rather foul, her pulse quick, and her bowels sluggish. She was of a very nervous temperament.

She could give no satisfactory account of her complaint, at least of its cause or commencement. She had always enjoyed tolerable, but never robust health, and from her youth had lived a very quiet, though rather confined, life, as a lady's maid. She was unmarried, and had never a venereal affection, nor ever taken mercury. The discharge had established itself gradually, and was at first thought by her to be a cold in the head. Its persistance for five or six weeks before I saw her, and the tumefaction of the nose, induced her to fear that it was something more serious, and to apply for my advice.

The case wore the aspect of chronic inflammation of the Schneiderian membrane, and probably of ulceration of some deeper portion of it. Under this impression, I directed the application of two or three leeches to the left side of the nose every third or fourth night—injections of warm water, followed by a solution of sulphate of zinc, and washes of that nature—gentle aperients and such remedies as would improve the secretions—and a course of sarsaparilla. These means were apparently attended with a good effect. The discharge diminished, almost disappeared—the tumefaction of the nose diminished too—the pain was no longer felt—and the general health improved. But still the patient was far from strong, and in consequence of her age, and her infirmity, her mistress pensioned her off, and, in the Autumn, she went, for change of air, to her native place, in Monmouthshire.

The weather was at that time very bad, and she suffered greatly from the wet and cold. She soon grew worse, and, after remaining for two or three months in the country, she returned to town.

A striking alteration had occurred in her appearance. She had lost much flesh, had the anxious appearance which betrays serious disease, was sallow, with a disposition to a feverish flush upon the cheek, was feeble in her gait, and rather hurried in her manner. The pulse was extremely weak and frequent, the tongue somewhat loaded, and the bowels disposed to be confined.

The swelling on the left side of the nose seemed to have rather increased than diminished, but was still much less than when she first came to me. The discharge had augmented materially. From the left nostril, it was purulent, but rather bloody, profuse, and offensive; from the right nostril there was also some, but it was yellow, and more free from odour. On inspecting the nares, nothing more was visible than the redness and tumefaction that were perceptible at first.

I concluded that the ulceration of the Schneiderian membrane had extended to the bones high up in the nares, and anticipated considerable mischief. It would be useless to particularize all the remedies that were employed—they mainly consisted in the iodide of potassium—sarsaparilla—the mineral acids—regulation of the bowels, &c.—and injections of various descriptions. They appeared neither to do good nor harm, for the symptoms continued for some six weeks or two months with little alteration, save that the patient *looked* worse and *felt* so.

At the expiration of that period I observed a tremulousness in the gait, which seemed to pass the limits of any ostensible debility—and there was an absence in her manner, and a defectiveness of memory which attracted my attention too. She no longer complained of the discharge being offensive to herself, but inquired very earnestly whether it was so to me. Her pulse was more quick, her skin rather warm, and there was a sort of low fever which forbade all medicines of a stimulating character. I had not seen her for ten days or a fortnight, when she came to me for the last time, accompanied by her sister. She had difficulty in entering the room, her steps were so unsteady, her regards so wandering, and her ideas so uncollected; in fact she was in a state of low delirium. Her pulse was rapid, and, as it had long been, the skin warm; the tongue was foul. She had lost a large quantity of blood from the nose that morning, and had been worse since the hæmorrhage.

Of course I sent her home immediately, gave such prescriptions as I thought necessary, and called to see her next morning. She was then comatose, but still the skin was *warm*, and the pulse though weak in the extreme was rapid. The pupils were dilated, and insensible to light—the limbs were not moved when gently pinched. She lay in this state for six and thirty hours, and then died.

*Dissection.*—I was kindly assisted in my examination of the head, by my pupils Mr. Barrett, and Mr. Ballantine. The idea of the case which I had formed was this:—*that*, ulceration of the Schneiderian membrane had extended to the æthmoïd bone, the upper parts of which were probably carious—that the cribriform lamella was implicated, or at least had been the means of carrying inflammatory action to the membranes at the basis of the brain, and that effusion of serum, lymph, or pus had occurred in their vicinity.

On removing the skull-cap, the veins of the pia mater on the surface of the hemispheres were found to be much congested, and there was some arterial injection too. The substance of the brain presented those dark spots on section, which mark a loaded state of its venous system, but otherwise the general texture of its upper part seemed unaltered.

The ventricles were greatly distended with turbid sero-purulent fluid, in which were flakes of lymph in considerable quantity. The septum lucidum was almost totally destroyed. The walls of the ventricles were softened to the depth of a line or more, and the softened substance, as well as that immediately beyond it, was marked with punctuated and slightly ramified injection. The effusion extended into the third and fourth ventricles.

The principal mischief existed in the anterior part of the left ventricle. The surface of the corpus striatum was ulcerated, and softened; greyish in colour, and marked with punctuated injection.

The anterior horn of the ventricle opened into an ulcerated cavity in the anterior cerebral lobe, capable of containing a moderate-sized walnut. Flaky sero-purulent fluid was found in it. Its walls were greyish, softened, injected, and its floor was particularly softened, ulcerated, and in a point destroyed, in the situation of the pars perforata antica.

This led to an examination of the basis of the skull and cerebrum.

Superior to the arachnoïd of the latter, stretching back from the pars perforata antica on the left side, was sero-purulent fluid and lymph. This deposit extended along the sub-arachnoid cellular tissue to the medulla oblongata and cord, which, so far as could be seen, were surrounded with it. From the circumstances attending the examination, it was impossible to open the spinal canal. This sub-arachnoïd effusion was limited to the basis of the cerebrum and to the medulla.

Attention was now drawn to the left æthmoïdal groove (the upper surface of that cribriform lamella) by a good-sized *drop* of pus which stood in it. This was found to be *in* and *on* a large foramen, through which one of the branches of the olfactory nerve had passed into the nose. On breaking up the cribriform plate, the pus was found to communicate with purulent matter situated in the upper part of the nasal cavity, besmearing its Schneiderian membrane, where that was not destroyed, and walls. The bone forming the latter was more or less exposed, dark, and carious. The æthmoïdal cells on the left side were laid open into the nasal cavity by caries, filled with pus, and partially broken down. But the os planum remained entire, the barrier between the nose and eye was left, and the latter organ was intact.

To revert to the superior surface of the cribriform lamella and the drop of pus seen on it. The dura mater and arachnoid on the former were sound. This pus was found, on inspecting the brain, to belong to a small abscess *in the substance of the left olfactory bulb,* which lay on the cribriform lamella in that spot, and received, through the opening by which the pus communicated with that in the nasal cavity, one of its nervous filaments.* The olfactory bulb, then was hollowed out into a small abscess. From this, there passed back *along* the *nerve* and *above* the *arachnoïd,* a layer of lymph. On reaching the spot where the nerve has its three-fold connexion with the brain, the lymph held on its course above the arachnoid towards the medulla oblongata, as I have already mentioned.

But the "root" of the olfactory nerve which connects it with the anterior cerebral lobe, was grey, injected, softened, and partially ulcerated, and this was *the* defective and destroyed point in the floor of the ulcerated cavity of the anterior cerebral lobe, which has been described already.

Such are the main features of this rather remarkable case. I think there can be little doubt that the disease ran this curious course:—inflammation of the Schneiderian membrane in the upper part of the left nasal cavity,† led to exposure and disease of the æthmoïd cells on the one hand—to extension of inflammatory action along an olfactory filament or filaments to the olfactory bulb on the other hand. This inflammation produced abscess in the bulb. The inflammation extended back along the nerve to its root, occasioned softening or ulceration there, and from that invaded the anterior lobe of the cerebrum, where it gave rise to an abscess. This opened, by ulceration, into the left ventricle, inflammatory action was set up in *its* walls, they were softened or in part destroyed, increased effusion was poured out, and distended not only the lateral ventricles, but the third and the fourth also.

Starting from the same point, the root of the olfactory nerve, the inflammation and consequent effusion took another route, that of the sub-arachnoïd cellular tissue, along the basis of the brain to the spinal cord.

I should state that my friend and colleague, Mr. Tatum, examined the cerebrum after its removal, and coincided perfectly with the view that has been stated. The preparation itself, though put in the strongest alkohol, was unfortunately so softened by its rough and clandestine removal from the body, that it would not bear the pressure of the dish, and was soon worthless.

This case bears some analogy to those which have at various times been published, in which disease beginning in the tympanum, or internal ear, or mastoïd process, has extended to the brain itself. But there is this considerable difference between them. In the latter cases the temporal bone being diseased, the dura mater and arachnoïd suffered from their connexion with it, and these membranes propagated the inflammation to those immediately investing the brain, and to the brain itself. In the case, however, which I have related, the dura mater and

---

* This phrase implies the passage of the olfactory nerve, from twig to trunk, *towards* the brain.

† I should observe that the right on examination was sound, at least the bone was so.

arachnoid covering the upper surface of the cribriform lamella was sound, the cribriform lamella itself was sound too, and the disease had extended along a nervous cord, not to a contiguous, but comparatively remote and deep part of the cerebrum.

The case most analogous to this, in point of principle, is where malignant disease of the eye extends along the optic nerve to the brain.

Independently of any interest that it may derive from singularity, this case has a practical one of greater value. It points out a danger to be held in view in inflammatory diseases of the nasal cavities. Nor is that danger to be looked on as altogether chimerical. The following instance appears to be one in point.

*Case.* In the course of last year, I saw a lady, about thirty years of age, who was affected with common vesicular polypi in each nostril. They were of tolerable dimensions, had descended into the inferior meatus, and did not seem to have a more elevated origin than such polypi usually have. The lady was unmarried, and of a very nervous temperament. She had not, I believe, had the polypi removed before.

I seized and extracted without difficulty, and with little force, some vesicular polypi from each nostril. It is a common recommendation to tear away a bit of the spongy bone from which the polypus grows. This, however, was not done in the present case, nor was the violence employed *so* great as is customary.

That evening the lady was attacked with shivering followed by fever, pain in the forehead, flashes of light before the eyes, and some confusion of intellect. It was necessary to apply leeches, and to resort to strict antiphlogistic treatment, before the symptoms were removed; nor could this be done in less than a week or ten days.

This case, coupled with the fatal one I have narrated, would lead a cautious surgeon to pause before he pronounced even so trivial an operation on the nose as the avulsion of a simple polypus, totally free from hazard. And they tend to throw doubt on the propriety of the direction, to endeavour to tear away a piece of bone.

I had intended to have offered a few observations on chronic inflammation of the Schneiderian membrane—a complaint both common and troublesome. But these remarks have already extended too far, and I cannot venture to intrude longer on the reader.

---

### DR. FLEMING ON ABSCESS BETWEEN THE PHARYNX AND THE SPINE.*

DR. Fleming relates two highly interesting cases, and adds many observations, illustrative of the occurrence of acute suppuration in the loose cellular tissue behind the pharynx. We shall take the first case.

*Case.* It was that of a boy. His age was three years and a half, and in appearance he was healthy. The premonitory symptoms of his attack, at first mild, after about thirty-six hours, assumed most intense severity, and without unnecessarily particularising their progress, it may be stated, that the most aggravated form of high inflammatory fever set in, principally engaging the cerebral organs, and requiring the most energetic treatment to combat it. On about the fourth day, convalescence appeared established. But from day to day a peculiar fixed position of the head, and stiffness in the neck, now attracted

* Dublin Journal, March, 1840.

attention. The head was drawn back. The muscles, at first tense, became completely and permanently rigid, and the movements of the head painful, and remarkably limited. Soreness in the throat was complained of, and also great difficulty in swallowing, at times accompanied with violent spasmodic efforts. There was no cough, and the voice remained perfect. The articulation became remarkable—the words being as if drawled out with pain and difficulty, and at times perfectly unintelligible.

Repeated and careful examination of the fauces and neck could not detect any apparent local cause for those symptoms, which, with varied degrees of intensity, advanced, producing equally alarming constitutional disturbance and debility. The treatment adopted was principally with the view of promoting the absorption of any fluid effused in the cerebrum, and consisted chiefly in the exhibition of mild mercurial alteratives, and the application of counter-irritants to the region of the occiput.

"On about the tenth day, the symptoms had reached their acmé; the child, emaciated and weakened, had no relish for food, and appeared to drink merely to allay thirst, the efforts at swallowing being convulsive and painful. He was now in a perfect state of somnolency, regardless of every thing about him, when accidentally, whilst sitting beside his bed, I perceived that *position* most remarkably influenced the severity of the prominent symptoms. Stupor in the recumbent posture, almost amounting to perfect coma, in the sitting, or even semi-erect, resolved itself into a comparative sensibility. Respiration slow, laboured, and stertorous, or rather roaring, (as described by the attendants on the child,) in the former position, became comparatively tranquil in the latter, and a pulse, in the one, ranging only a beat or so above forty, in the other, assumed a more natural character. Again, fluids were more frequently darted convulsively forwards through the nostrils or mouth, than passed into the stomach, or were ejected, as in the act of vomiting, and the recurrence of the symptoms of cerebral compression took place on returning to the recumbent posture, which for the last three days had been almost the permanent one.

I now considered that this relation of symptoms might still be caused by mechanical obstruction in the pharynx, although repeated examinations on former occasions did not lead me to this conclusion. An additional obstacle presented itself in the fixed position of the jaws, so that it was only by considerable force I could so far separate them as to admit of even getting my little finger between them. On forcing it back, I accidentally, but distinctly, felt a tumefaction beyond the base of the tongue, giving, as well as a compressed finger could indicate it, a sense of yielding. To get a view of it was utterly impossible. The soft palate and uvula were easily discernible, but the depression of the tongue gave so much pain, and the separation of the jaws was so very limited, that further investigation was totally out of the question. Indeed, in addition, the evidence, even from touch, was necessarily momentary, from the severe paroxysms of dyspnœa attendant on the examination.

Although I had never heard of, nor witnessed a case of the kind before in children, it at once occurred to me, that this might be an abscess at the back of the pharynx, mechanically producing the above symptoms, and having stated this as my opinion to the family, the assistance of Dr. Crampton and Mr. Cusack was immediately procured. After a patient, though extremely unsatisfactory examination, they coincided in opinion with me as to the presence of a tumor in the situation alluded to, and it was determined that I should perforate it with an explorator which I had provided for the purpose, with the view of ascertaining its actual nature,—a doubt existing on this head, not alone from the extreme firmness of the tumor communicating a very indistinct sense of fluctuation, but also on account of its probable anomalous nature from the previous acute and present chronic cephalic symptoms. With every necessary precaution I accomplished this object, though with considerable difficulty, and to my great gratification, witnessed the sudden gushing forth of a large quantity of healthy purulent matter. The whole features of the case were almost instantaneously altered. The somnolency was removed, deglutition was facilitated, and more cheering prospects manifested themselves. Nourishment was freely given throughout the day, and quinine administered in small and repeated doses."

The symptoms were returning in the evening, when Dr. Fleming found, on examination, that the abscess was again filled and the opening closed. He introduced a carefully protected sharp pointed bistoury, into the site of the opening, and freely enlarged it downwards. The relief was instantaneous. He directed the trunk of the child to be elevated as much as possible, and the head depressed. The night was passed comparatively tranquil; the quantity of matter which escaped through the mouth was considerable, largely staining the pillow. The next day, the boy was able to play with his brothers, and subsequently his improvement was progressive, though slow.

The next case is very similar, save that the child was an infant at the breast, the abscess below the level of the tongue, and the fluctuation more obscure. A peculiar instrument, therefore, became necessary. It consisted of a trochar about four inches long, one extremity of the cannula being slightly curved, the other with a ring on its upper surface to receive the fore-finger; into this cannula was passed a jointed stilette, with, at its opposite extremity, a ring for the thumb, and a moveable screw to graduate the projection of its point. The head of the child being firmly supported, Dr. Fleming passed the forefinger of the left hand towards the back of the pharynx, there resting the point of it, and guiding the armed trochar with the concealed stilette along it, accurately fixed it on the tumour, pressed forwards the stilette to its limited mark.

Dr. Fleming has not seen any similar acute case in the adult. But some have been related—one by Sir Astley Cooper—one in the Dictionnaire de Medicine et de Chirurgie Pratique, under the head "Pharyngotome et Pharyngotomie." In all these cases, the abscess was formed before it was suspected.

Dr. Fleming makes some lengthened observations on these cases, but, though instructive, we cannot go into them. We think the one case we have quoted, together with the following summary of his opinions, will enable our readers to see their bearing in a sufficient degree.

"I consider this affection of the throat in children, when *acute* in its progress, as, often, an inflammation of a lymphatic gland situated at the back of the pharynx; an inflammation extremely rapid in its progress to suppuration from its particular position; that I would watch for it during the period of difficult dentition, and in the several cutaneous affections or diseases of the gastro-intestinal mucous membrane to which children are liable; and that I would consider as strongly pathognomic of its presence the following symptoms :—

Fever, more or less sthenic in its character according to the peculiarity of constitution of the child, is always present, and, I think, precedes the development of the local symptoms.

These local symptoms are premonitory and essential.

The *premonitory*, indicative of *local* uneasiness, but yet common to all affections of the throat; complained of, or otherwise, according to the age of the child, and on examination, not accompanied with proportionate visible lesion. The *essential*, often very suddenly supervening, and indicated by derangement of the cerebral, circulating, and respiratory systems, alternating with the comparatively healthy condition of those systems, according to the alteration in the position of the individual.—Fixed and retracted state of the head, with rigidity of the muscles at the back of the neck, and more or less locked state of the jaws. —Painful deglutition, impossibility of swallowing solids, and fluids convulsively darted forward through the mouth and nose.—Repeated acts of deglutition without the presence of any fluid in the mouth, and, on examination of the fauces, a firm, projecting tumour felt beyond the base of the tongue, and if seen, presenting a smooth, rounded, highly vascular appearance behind the soft palate usually occupying the median line, but occasionally inclining to either side. These *essential* symptoms accompanied with the ordinary characteristics of suppurative fever."

When such symptoms are present, an abscess should be looked for, and an opening (if there are any indications) should be cautiously made.

---

### Plastic Bronchitis treated by Mercury.*

Dr. Cane has published in our contemporary a valuable paper, on Plastic Bronchitis, or Bronchial Polypi. The concluding observations, short as they are, exhibit its object.

"The facts, I conceive, derivable from the cases now presented to the Profession are, that plastic bronchitis is a disease *sui generis*; that the sputa are essentially distinct from those bodies termed polypi, and which are no more than coagula, freed of the colouring matter of the blood; I allude to those found in the heart; and those sometimes ejected from the bronchi of patients who have had hæmoptysis. With those they have no character in common, except that of shape, being moulded in tubes of a like form; indeed it is unnecessary to dwell upon the differences, for the preparations, as exhibited on the plate, will at once display them. That as far as the two cases, now published, go, they are not indicative of phthisis, and that mercury is a certain remedy for cure, are facts the more valuable, because of the concurring testimony afforded by the valuable case of Dr. Corrigan."

Dr. Corrigan's case happens to be more concise than Dr. Cane's, for which, and for no other reason, we cite *it*. To Dr. Cane is due the merit of the paper.

*Case*. On the 14th August, 1839, Mr. A. aged 40, called on Dr. Corrigan. "As he entered my study, his aspect seemed that of a man in the last stage of valvular heart disease: his countenance was sunken and anxious, his lips were bluish, and his respiration so laboured as to be almost painful to look at; each inspiration was accompanied with a wheezing, so loud that, at first, I thought it was produced in the larynx, but his voice was unaltered. He told me that, notwithstanding his apparent distress of breathing, he was at that moment comparatively easy; that at times the distress of breathing became most severe. On several occasions within the last three weeks, he had attacks of suffocation, coming on in the course of the night, lasting so long as half an hour, and as he described them, threatening almost death. Sometimes, for hours, he has been obliged to sit up, with the window open. These fits terminated in expectoration. He has no palpitations; his appetite is good, and his bowels are regular, he attributes his illness to cold, as its commencing cause, caught about twelve months since, when, after exposure on a coach, he got cough; then in the spring, influenza; and within the last three weeks the suffocative attacks. On examining the chest, I found the sounds of the heart quite natural, and the sound on percussion over the chest good; but on applying the stethoscope under the right clavicle, my attention was at once suddenly arrested by the great irregularity of the respiratory murmur. At one moment the respiratory murmur was very loud, and the next instant it was nearly inaudible. The clear wheeze, above noticed, (*rale sibilant*) was immoderately loud and piercing under the right clavicle, but on tracing upwards with the stethoscope, it became less loud as the stethoscope

---

* Dublin Journal, March, 1840.

approached the larynx. These singular varieties in the respiration made me suspect the existence of aneurism or tumors, &c. pressing on the larger bronchia, but I sought in vain for any sign of their existence. I then desired him to cough very freely. He coughed, hard and with a bronchitic ringing, for some moments, and after some efforts expectorated four or five bronchial polypi or moulds of lymph of the bronchial tubes. Of these plastic concretions, one was as thick as a small-sized goose-quill and about an inch and half long; several were much smaller in diameter, but longer, and all were white, opake, and remarkably tough. The expulsion of these plastic secretions was immediately followed by a very remarkable change in the state of respiration. The respiratory murmur instantly became suddenly loud, and equal in both lungs, and the wheezing ceased, nor could he again, by coughing or by any effort, reproduce it. The nature of the case was now clear: some of the bronchial tubes had taken on this plastic secretion, and as this formed each successive night, it blocked up the bronchial tubes, until, at last, the obstruction in these tubes rose to such a height as to bring on impending suffocation. From this he only got relief by fits of coughing, which dislodged the secretion, and then there was an interval of ease until the secretion began again to be formed."

Dr. Corrigan directed the cessation of the antispasmodics, &c. and ordered him 10 gr. of hyd. c. magnesia three times a-day, with full doses of aqua kal. caustici, and desired him to inhale, twice a-day, the steam of water in which conium leaves were infused. In five days the mouth grew sore, the plastic secretion ceased, and on the 26th inst. the patient was well.

---

### SLOUGHING OF THE UVULA, FROM CATARRH.*

Dr. Berkeley, of the Philadelphia Dispensary, had under his care a girl, ten years old, who had laboured under a slight catarrh, for a few days. A remarkable change in her voice induced her parents to ask medical advice. Upon examining the throat, the fauces were found so much swollen as nearly to close the entrance into the œsophagus; while the uvula was thrown forward against the roof of the mouth, much enlarged, and sloughing at its extremity.

The girl complained of no pain whatever, and seemed to suffer only from an inability to swallow any thing but fluids. She was directed to use stimulating gargles, till there was a return of sensation in the parts, and the uvula was touched four or five times a day with a solution of nitrate of silver, (iv. gr. to the oz.) On the third day a considerable portion of the uvula was thrown off—the swelling of the fauces had been much reduced; and the sensibility of the whole surface so far restored as to render the use of stimulants no longer necessary. A mild astringent gargle was then directed, which was continued till the eighth day; when the patient was discharged, cured; no symptoms of the affection remaining, except a slight difficulty in pronouncing a few words, in which there were many consonants.

---

### SKETCHES OF FRENCH SURGERY AND SURGEONS BY AN AMERICAN.

To those who are in love with Continental Schools of Practical Surgery, the following sketches by an American Physician, Dr. Harlan, published in the Medical Examiner, of Philadelphia, may not be devoid of profit. We would

---

* Medical Examiner. No. 25, Vol. II.

earnestly direct attention to his observations. There is a growing disposition in this country, a disposition, unfortunately fostered by some, to look with admiration on Foreign Medicine, and more particularly on Foreign Surgery, and to imitate that mere *artiste-like* skill, mechanical dexterity, and disregard of the *scientific* treatment of disease, which have hitherto contrasted slowly with the useful and honourable, though not the showy characters of English practice. Let us hear from an eye-witness what Parisian Hospitals and Parisian Surgeons are.

" Parisian hospitals and French surgery might be presumed, *à priori*, to be the first objects of attraction to a practical surgeon; but I cannot but confess that a longer acquaintance with them, a more extended course of investigation, and a more familiar intercourse with their most eminent teachers of surgery, have in no small degree lessened the admiration with which I once viewed the éclat generally attributed both to the men and the institutions. It is true, we cannot too much admire the long-continued and laborious application by which they have attained perfection as anatomists, and the consequent manual dexterity in operations, so universally admitted as a distinguishing characteristic of French surgeons,—and here their dexterity or superiority ends. Not only so: this dexterity itself has been obtained at the expense of principle and at the expense of life; thousands are annually consigned to a premature grave by operations not always necessary to be performed at all, or improperly timed, or performed in cases that must terminate fatally, with or without operations. The mortality occurring at the Hôtel Dieu, perhaps one of the best, is absolutely frightful in amputations alone; the surgeons admit a loss of ninety-five per cent; and one of the internes admitted, that during his residence for one year at the Hôtel Dieu, not one case of recovery occurred after amputation! I esteem M. Roux, the surgeon-in-chief of this extensive institution, as a personal acquaintance, and would not heedlessly detract from his hard-earned reputation; and in thus alluding to the results of my own personal observations, I have the interests of science only in view."

Here is a sufficient answer to the assertion, that great operators are not necessarily prone to perform operations that might by caution and skill be dispensed with. A knowledge of human nature would suggest the contrary, and experience confirms it. Men will be fond of what they are conscious of excelling in, and there is something particularly attractive in the éclat of capital operations. The fruits of studying operations as *the* great aim and object of a surgeon, are perceptible in France, and will be visible ere long, unless a strong resistance is offered to such a spirit, in England.

*M. Roux.*—" M. Roux possesses a grave, earnest, and decided character; but, like most others of his profession here, he is over-fond of displaying his manual dexterity. I have heard him beseech a patient to submit to an operation, as if it was the greatest favour conferred upon the operator; the operation once performed, the patient is pretty much consigned to his fate, for the after-treatment of French surgeons I consider little better than no treatment at all. The constitutional demands, the habit, diathesis, or idiosyncrasies of the patient, are almost universally and entirely overlooked, and hence, together with the foul air of the hospitals, the dreadful mortality of these pest-houses. On the very first *coup d'œil* of the wards of these hospitals, nothing but disastrous consequences could be anticipated from one hundred to one hundred and fifty human sufferers, crowded together, side by side, and exhaling each the noxions effluvia peculiar to the gorgon form of diseases which afflict the inhabitants of rooms constructed on the worst possible principles for the purposes intended, and in which ventilation was not thought of, and where classification of disease has never been attempted."

" As a lecturer, M. Roux is animated, though by no means eloquent. During a private interview I held with him at his house the other day, he complained seriously, and lamented the state of French surgery of the present day in comparison with its former state; no one surgeon now, he said, could obtain half so many *operations* as formerly,—there were so many hospitals, and then each institution was 'si partagé,' 'si isolé,' there being eight or ten surgeons to each, and then almost every department of surgery being pursued in particular by some surgeon of eminence, that but few opportunities comparatively were left now days for the surgeon of a general hospital to show his skill. Only seven cases of lithotomy occurred in his own wards last year. Thus the diseases of the eye, the ear, hernia, club-foot, affections of the bladder and urinary organs, venereal disease, &c. &c., have each a hospital devoted exclusively to themselves. M. R. is on the most familiar terms with his patients. In going his daily rounds from 7 to 9 A. M., he has always something funny, encouraging or coaxing, to say to them all; for one he has a poke in the ribs with his finger, for another a box on the ear, &c. I have seen him make a convalescent reel with a blow on the side of his cheek, for asking him for something good to eat—all in the best possible humour. He sometimes becomes very affectionate, and kisses a patient on whom he has just inflicted a severe operation; and this salute, the students say, always prognosticates the death of the sufferer.

M. Roux mentioned to me Physick, Warren, and Mott, as the only American surgeons with whom he was acquainted; and, judging from them, said he wondered at the large and rapid fortunes that were accumulated by American surgeons—a consummation which he feared would never attend the efforts of Parisian surgeons.

In person, M. R. is rather beneath the ordinary stature, of a sanguino-phlegmatic temperament—features blunt and ill-favoured—one eye projects from its socket, and has a cast in it. When earnest in discourse, his countenance is especially contorted."

*M. Blandin.*—This gentleman is also attached to the Hôtel Dieu. He has lately been coaxing his *stumps* to heal by the application of warm air after amputations, the stump being enclosed in a glass caisse, and air heated to the natural temperature of the body caused to pass constantly through it, is left without further dressing. Healing by adhesive inflammation, until within the last few years, was unknown in Parisian hospitals; from time to time some of the surgeons have attempted the adhesive process, but they are by no means fully aware of its importance. These stumps appeared to be granulating well, which may probably be accounted for by the simple circulation of air thus artificially produced.

*M. Velpeau.*—"M. Velpeau has been most unfortunate in the loss of patients in La Charité, in which hospital the mortality has been greater than in any other establishment. In addition to the usual causes which have gained for this hospital so mortifying a distinction, there has prevailed during the last winter and present spring an erysipelatous diathesis, which has desolated its wards, so that a puncture of a nail, the simple operation of extirpating a small ganglion from the neck of a healthy subject, the operation for the radical cure of varicocele, and amputations of all kinds, fractures, &c. I have seen terminate fatally in numerous cases; and yet M. Velpeau continued his operations, and appeared astonished himself at their want of success, and is far from taking advantage of the best means of averting the evil; his constitutional treatment is worse than nothing. A patient was admitted with a wound in the heel by a nail; erysipelatous inflammation followed, and was continued to the ankle-joint, producing suppuration, irritation, and death. No measures were resorted to in order to rally the powers of a broken constitution; and in the autopsy and lecture on this subject, M. V. expressed his inability to explain his want of success in the treatment of the case, and viewed the death of a patient from the simple puncture of a nail as an opprobrium to surgery! But human life, it is said, is of very little account with French surgeons. M. V. operated five times successively, for disarticulation of the knee, and in every case his patients died.

But M. V. is a most dexterous and fearless operator; and if his hospital was more fortunately constituted, would doubtless be more successful. I have seen him twice remove tumours involving large portions of the lower jaw, with complete success; but he frequently operates needlessly and recklessly: he is more anxious to display his dexterity than to cure his patient. As a lecturer, M. Velpeau is quite too eloquent, the idea being lost in the midst of verbiage; hence, M. Lisfranc has given him the soubriquet of 'le Paroquet,' and sometimes he calls him 'the Blacksmith'—M. V. having been educated to that trade—of which, however, he is in no manner ashamed, frequently alluding to the period of his life when he was 'un maréchal.'"

Amongst other things calculated to ensure success in practice at home, the English surgical student in France may acquire, we see, gentlemanly manners. One surgeon gives nicknames to the other. But that is nothing to what follows. M. Velpeau's address is anything but that of a gentleman. His personal appearance is vulgar, and his aspect forbidding; but his ugly countenance is agreeably relieved by a noble and expansive forehead, to which "oasis" he undoubtedly owes all his celebrity. He has a most disgusting habit of using the long, thin hair which covers his head as a mop, on which to wipe his hands when soiled by some putrid piece of pathological anatomy, on which he is expatiating before his class, every few minutes passing his fingers through his hair! For the politest people in the world this is pretty well.

The following anecdote of Velpeau and Heurteloup is *piquant.* The admirers of the Baron in this country will be taken aback by it.

"Two of my friends, an English and American physician, called recently on M. V. at his office. On being ushered into an adjoining apartment, they overheard M. V. and another person altercating in the highest tone of voice; (this visiter proved to be Baron Heurteloup.) They could distinctly hear M. V. address the Baron in the following manner:—'Mais, M. le Baron, vous savez que vous êtes un grand menteur.' When the Baron appeared to become furious, demanding to know what he meant by such an assertion, M. V. continued—'Soyez tranquille, soyez tranquille, M. le Baron; mais vous savez, entre nous, vous *savez* que vous êtes grand menteur!' Here the subject was dropped; M. le Baron, (who, by the way, I conceive to be much of an adventurer,) not considering it any insult to be called by a name which he himself is very liberal in conferring on his confrères, especially as he was not aware that any third person was a witness to the scene."

In the case of an old man, with fistula in ano, an incident occurred which shewed M. Velpeau's fondness for cutting. After the ordinary section, the surgeon amused himself in trimming the cut edges of the wound, very deliberately, when, all at once, a large projecting mole on the back of the patient caught his eye. It was too tempting a bait. He immediately transferred his scissors to it, and incised it at its base, very much to the discomfiture of the patient and merriment of the class, one of whom remarked that M. V. *cultivated* a patient's *acquaintance* very much like the gardener cultivated his plants, by lopping off all offensive parts and useless exuberances.*

*M. Magendie.*—"His spring course of experimental physiology commenced in the beginning of April. I seldom fail of 'assisting' at his murders. At his first lecture, a basket full of live rabbits, a glass receiver full of frogs, two pigeons, an owl, several tortoises, and a pup were the victims ready to lay down

* Medical Examiner, No. 28.

their lives for the good of science! His discourse was to explain the functions of the fifth pair of nerves. The facility was very striking with which the Professor could cut the nerve at its origin, by introducing a sharp instrument through the cranium, immediately behind and below the eye. M. Magendie drew the attention of the class to several rabbits, in which the fifth pair of nerves had been divided several days before. They were all blind of one eye, a deposition of lymph having taken place in the cornea, from inflammation of the eye always following the operation alluded to, although the eye is by this section deprived of all sensibility. Monsieur M. has not only lost all feeling for the victims he tortures, but he really likes his business. When the animal squeaks a little, the operator grins; when loud screams are uttered, he sometimes laughs outright. The professor has a most mild, gentle and amiable expression of countenance, and is in the habit of smoothing, fondling, and patting his victim, whilst occupied with preliminary remarks; and the rabbit either looks him in the face, 'or licks the hand just raised to shed his blood.' During another lecture, in demonstrating the functions of the motive and sensative fibres of the spinal nerves, he laid bare the spinal cord in a young pup, and cut one bundle after another, of nerves. The phrenological developments of the professor belie those of physiognomy. His skull is enormously broad between and behind the ears. Living dissection is as effectual a mode of teaching as it is revolting, and in many cases the experiments are unnecessarily cruel, and too frequently reiterated; but so long as the thing is going on, I shall not fail to profit by it, although I never wish to see such experiments repeated."

---

### Dr. Gerhard's Clinical Lecture on the Inflammatory Origin of Tuberculous Disease.*

Dr. Gerhard observes, that the serous membranes afford us the best opportunity of studying the form of tubercles at their earlier period, and from the same transparency enable us to decide, with tolerable precision, the vexed question of the inflammatory or non-inflammatory origin of tubercles. When the case is acute, he goes on to say, it is much more obviously inflammatory than in those which are slower. These acute cases are attended with considerable febrile excitement, which sometimes becomes violent, and not a few patients perish during the early period of the disease, from the immediate effects of the pleurisy, and not from phthisis. Upon examining the pleura in these cases, you will find that the semi-transparent granulations are surrounded by a red circle formed by the blood-vessels developed during the course of the pleurisy. The vessels are most numerous in those parts of the pleura where the tubercles are largest, and placed nearly in contact. The vessels are arterial, and are visible to the naked eye; there are, however, others much smaller, and only distinguishable by a microscope. These vessels evidently do not enter the tubercle, but merely surround it, and perhaps communicate with the investing capsule. The smallest granulations like the largest, are surrounded by the same circle of vessels, and are evidently secreted in the same manner. The pleura may be detached from a portion of the granulations, but others come off with it. This arises from the fact that the lamina constituting the pleura proper does not enter into their substance; it is only the intermediate, and, as it were, cellular coats of the pleura, which nourish the newly-formed structure.

Besides the pleura proper, the false membrane, or coating of lymph, effused upon and within the serous membrane, is often the seat of tuberculous deposit.

---

* Medical Examiner, No. 28.

This never takes place except when the false membrane contains numerous red vessels, and is evidently in a state of active inflammation. It scarcely happens that tubercles are found in false membranes, except in connexion with these evident marks of inflammation; where they are met with under other circumstances, the membranes have contracted firm adhesions with the pleura, and have lost their distinctive character of newly-formed lymph. The evidence of inflammation, then, is conclusive he thinks in these cases.

Dr. G. has selected the case of tubercles of serous membranes merely on account of the clear demonstration of the steps of the process which is furnished by a transparent membrane; but in the other tissues of the body the same rule holds good. In tuberculous disease of the follicles of the intestinal canal, particularly of the glands of Peyer, he has traced the same vascular development at the commencement of the disorder. The injection was nearly as intense as in the serous membranes; at least, the difference was not greater than is usually observed in the inflammations of these tissues. The inflammation was not secondary, because it was developed at the very commencement of the lesion, when the most minute points of tuberculous matter could be detected, and was by no means confined to the cases in which the granulations had attained a considerable size. In the lungs, in certain cases, the same inflammatory process is quite evident; but, in these organs it is chiefly confined to two varieties. In one of these varieties, you find a large portion of the tissue of a bright red tint, rather more vermillion than in ordinary cases of pneumonia, and thickly interspersed with semi-transparent granulations of a remarkably equal size, as if they had been secreted simultaneously; or, as it were, by the action of the vessels. In another variety, the lung is studded with nodules of a darker red, composed of one or more lobules and quite hard; these nodules are found to contain an immense number of semi-opaque granulations; apparently deposited in the vesicles themselves, and contrasting, by their dull white or yellowish tint, with the redness of the proper pulmonary tissue. In the lymphatic glands, in the brain, especially its membranes, and occasionally in other organs of the body, the same coincidence of unequivocal inflammation is often met with, and nearly always the tuberculous disease is of the acute form, and terminates very quickly.

The question is—must we look on this as common inflammation, or as specific?

" That it is a true inflammation is very clear, for it not only presents its usual anatomical characters, but the disease evidently arises from the same causes as commonly give rise to inflammation, and follows the same order of symptoms; but, it is equally evident that the majority of cases of inflammation of organs are not accompanied by a secretion of tubercle. Hence, there is certainly some other cause at work, the intimate nature of which is unknown."

An interesting lecture.

---

### Dextrine Bandages for Fractures.*

The following is Velpeau's method of application in fractured os femoris: the limb is in the first place enveloped in a common roller; a piece of bandage is then crossed over the ankle, for the purpose of extension, and over these is placed another roller, thoroughly impregnated with the prepared paste; compresses of coarse paper, similarly soaked, are applied over the fracture, and other prepared rollers applied over these,—coarse pasteboard compresses, softened by

* Medical Examiner, No. 24.

similar soaking, are also applied, and kept in position by other rollers. The extension is now made by tying the bandage from the ankle to the foot of the bedstead, (which are all of iron in these hospitals,) and counter-extension is made by a strap passed between the thighs, and made fast to the bedstead above the head. The bandages harden in a few hours, and the limb is incased in a solid coat of mail.

Experiments have been made with numerous adhesive materials, such as starch, glue, paste, gum, &c., as substitutes for the 'Dextrine' bandages now used exclusively at La Charité, all the others having been proved less effectual; gum arabic bears the nearest approach to it, but this is too expensive. The 'Dextrine' is made by boiling starch in dilute nitric acid, and when separated from the liquor and dried, the powder costs about thirty cents a pound. One great advantage which this powder possesses over starch is, that it may be made into a paste with *cold* water, the former requiring boiling water. A very excellent and cheap substitute for linen or muslin bandages has been found in coarse paper, cut into suitable length and breadth, and soaked in this paste previously to their application; there can be nothing more effectual than a Scultet's bandage thus applied,—a method of economizing by no means beneath the notice of Parisian hospital surgeons, especially since a recent ordonnance of government, which obliges them to substitute paper for linen compresses—the savings thus accruing to be devoted to the gilding of that magnificent temple, the Madeline church! A more detailed account of this 'Dextrine' may be found by referring to 'Orfila's Chemistry.'

Dr. Harlan speaks very favourably of the results. The bandage answers better for fractured thigh than for fractured leg. It is very little troublesome to the surgeon, one single application being generally sufficient, especially if, before applying the bandages, sufficient time is allowed to permit the subsidence of all tumefaction; for this purpose, the patient is permitted to rest in an easy position for three or four days, when, if the bandage does not become loosened by the subsequent atrophy of the limb, a single application will suffice for the cure,—but even in case of reiterated application, it is less troublesome than the usual method.

---

### STATE OF THE PROFESSION IN SPAIN.*

The Spanish doctors seem to be bad enough, yet not so bad as they have been represented. There is an old pictorial proverb on the devil, which is not inapplicable to them. They possess great respectability, and, while the priesthood have been retrograding, they have at least remained stationary. This is something in Spain!

One of the principal causes of the disrepute in which the Spanish faculty is held by their brethren, is the very small compensation they are said to receive for their services. Their compensation, measured by a foreign standard, is truly moderate and inadequate, but in reality is not so much so as believed; for, in the first place, their fees being low, they are oftener employed; and, in the second, they are paid in cash, so that they do not, from having long accounts and numerous charges against their patients, appear to be in the receipt of very large incomes, and yet have, in fact, very small ones, as is the case in the United States and other countries, where accounts are kept for professional services rendered. Moreover the Spanish physicians enjoy very great advantage in this respect, that they have none of their practice taken away from them by quacks,

* New York Journal of Medicine, No. II.

and the unlimited sale of nostrums; for neither the former nor the latter are permitted—laws existing for the suppression of all such nuisances to the profession and to the public.

"From the Spanish faculty partaking in a great measure of the reserve peculiar to the nation, I was not able to learn as much of them as otherwise I should; but, from what I could observe, they may be said to be polite and dignified in their manners; as well, if not better, informed than any other class of men in Spain; and in their profession much more practical than theoretical; being little given to writing. In this respect, they are extremely singular, for an original professional work is nearly unknown; and even to periodicals they seem to have an antipathy; for, according to what I could learn, the only one published is in Madrid, and this has so limited a circulation that it was impossible for me to obtain a single copy. For recent information they rely almost exclusively on French publications, and chiefly on the periodicals published in Paris. The only English works I met with were those of Cullen and other medical authors of about the same period. These works were mostly translated into Spanish. Of those by American authors I met with none; and the Spanish physicians seem to be acquainted with few of even the most celebrated of them. Finally, in consequence of the perusal of French publications, Spain having been so often over-run by their armies, and many of the Spanish faculty having been partly, if not altogether, educated in the medical schools of France, their practice appears to be chiefly that of the modern French school, though it still partakes considerably of the ancient practice in Spain and other parts of Europe.—*Horner, in Bell's Med. Lib.*"

---

### Notes on the Treatment of Cholera with Acetate of Lead.

Dr. Graves, in a letter published in the London Medical Gazette, of Oct. 14, 1837, strongly recommends the treatment of cholera by the free use of the acetate of lead, and "implores the profession in every part of the world, where cholera prevails, to give his plan of treatment a fair trial." Influenced by this, Dr. Morehead, of Bombay, determined, when the opportunity should offer, to put the plan into execution.

During the month of May, 1839, fifteen cases of cholera were admitted into the European General Hospital, twelve were of sailors belonging to merchant ships in the harbour, and three, of children of the garrison staff. Of the twelve sailors, seven died and five recovered. The seven who died, were admitted into hospital many hours after the disease had been fully formed; in all, the pulse was imperceptible or nearly so; the skin was cold, damp, and livid; the features sunken; and in almost all, the purging had ceased. They were treated with stimulants, chiefly brandy, ammonia, sinapisms, dry heat, and frictions, and in several of the cases, where a return of the purging was apprehended, the acetate of lead pills were also exhibited.

Of the three children, two died, and one recovered. In the two fatal cases (3 and 4 years of age) cholera mixture and Dalby's carminative had been given before admission; the purging ceased, but, in both cases, convulsions succeeded by coma took place, and death quickly followed. In the successful case, the sinking of the pulse was never great, and the treatment consisted in mild mercurials, with quinine and opium, sinapisms and blisters.

In the other five successful cases, the acetate of lead was more or less freely used.*

---

* Transactions of the Medical and Physical Society of Bombay, Vol. II.

### CUBEBS SHOULD BE FRESH-POWDERED FOR USE.*

At a meeting of the Surgical Society of Ireland, Mr. Morgan made some observations on the mode of giving cubebs, which are not undeserving of attention. He commenced by remarking the diversity of opinion that obtains with regard to the efficacy of the medicine. He went on to say, that, supposing, of which there could be no doubt, that the *essential* oil was the active medicinal principle, the problem was easily solved. On looking into the history of the Java pepper, he found that it, like most other vegetable products, was largely adulterated, and shamefully prepared for medicinal use. The London and Liverpool wholesale druggists, who supply the apothecary with the drug, send it chiefly in the state of powder, having previously added a fair proportion of pimenta berries, and Turkish yellow berries, (that is, the dried fruit of the rahmnus catharticus,) and it is much to be feared also, deprived it of a large proportion of its essential oil. But, taking for granted all was right, no adulteration, no extraction of the oil, its being so kept in the powdered state by the apothecary, the essential oil is rapidly dissipated; and on looking at the cover of the containing jar, the oil is seen largely adhering. Those facts induced him to speak to Mr. Herron, the respected proprietor of our national medical hall, on the subject, and he fully and entirely agreed in the propriety of purchasing a mill, and grinding the whole pepper as the prescription came to his compounding department. The result was most satisfactory. That the recently ground cubebs contain a much greater proportion of essential oil, Mr. Morgan satisfactorily proved, by a simple experiment. He procured from different shops in town samples of powdered cubebs, among them one ground in London late in the past year, as also one recently ground in Mr. Herron's mill. He placed each sample separately, in several folds of bibulous paper, and subjected them to pressure. On examining the packets he found that those had in the powdered state, scarcely soiled the envelopes, while that *recently ground* perfectly saturated all the folds: he considered that the experiment also proved the necessity of dispensing the medicine in stoppered bottles; or, what would answer equally well, and pay the apothecary better, in waxed papers. (He here handed round the samples, stating that they spoke for themselves.) The thirst for adulteration was so great that they even tampered with the unground pepper; but in that state it was easily discovered. The pimenta berries want the foot stalk, or bilocular, and contain two seeds; the cubebs, one; the rhamnus catharticus, four. To prove the thing practically, Mr. Morgan selected twelve cases of gonorrhœa indiscriminately. He treated six with the recently ground cubebs, and six with the powders had from different shops; the result proved the decided superiority of the former.

These observations are not undeserving of attention. No doubt much of the want of success, so much complained of with regard to cubebs, may be due to adulteration or to staleness of the drug. We may mention that a very good form of cubebs is prepared by Mr. Adams, a chemist of Charles Street, Pall Mall, London. He has termed it "Balsam of Cubebs." The dose is thirty to forty drops twice or thrice daily.

---

### MR. CARMICHAEL ON THE ORIGIN OF MALIGNANT DISEASES.

We find in an interesting Lecture on Malignant Diseases, published by Mr.

* Dublin Medical Press, Feb. 12, 1840.

Carmichael in the Dublin Medical Press,* some observations in reference to his peculiar opinions on the subject, which are not unworthy of attention.

The doctrine, he says, which at present most generally prevails, is that they are morbid secretions, incapable of becoming organised, like healthy depositions, in consequence of unhealthy changes in the mass of blood. He argues against this. How, on this supposition, can we explain that a cancer commences in a point and acquires its volume by slow and progressive degrees? If it were a morbid secretion, it would not thus always have a minute origin and a gradual increase, but develop itself largely, and perhaps, at once engage the entire organ assailed. Again, it is acknowledged by those who espouse this opinion, that inflammation has nothing to do with these foreign productions, although they attribute them to the depositions of unhealthy lymph, yet we do not find that lymph, healthy or unhealthy, is ever deposited without its usual precursor or concomitant, inflammation.

Again, although it might be granted that tuberculous or carcinomatous productions might be owing to the deposition of unhealthy lymph, arising from a morbid state of the blood, this theory will not account for the fact, that cancer may arise in the healthiest person, if any part of his frame is subjected to such external violence or continual irritation, as to injure the organization of that part. This is a fact that must be familiar to every experienced surgeon. How will this hypothesis account for the prevalence of cancer in the breasts and uteri of women at that period of life when the catamenia first cease, when they are no longer capable of child-bearing, and consequently, when these organs become useless appendages to the female system? But the fact is, that if the disease had *always* a constitutional origin, arising from some vitiated state of the fluids, not at present understood, we should never see operation attended with success. Mr. Carmichael admits the paucity of successful cases, but still contends that, were the disease constitutional, success should *never* happen. The following is his criterion by which to determine the propriety of operating or otherwise.

"There *are* indications which point out sufficiently well, when cancer has not a local but a constitutional origin, and therefore, prohibit in such cases any attempt at extirpation. These signs are a delicate sickly appearance, but above all, a peculiarly pale anæmic countenance, inclining rather to a bluish or leaden hue, as if the blood had not been duly oxygenated.

If a person with this description of appearance should apply to me for advice on account of a cancerous tumour, nothing would induce me to recommend operation, even, although the lymphatic glands in its neighbourhood, were totally free from disease, and the lungs perfectly sound. I should be of the same opinion, even though the tumour in question were attributed to external violence and not to any fault in the patient's constitution. For the accession of any tuberculous or cancerous tumour in a person of this peculiar bluish pale, or leaden colour, would indicate to me, that the disposition to the formation of such tumours was constitutional, and therefore, that any attempt to remove a *single one* by operation, or other means would be fruitless, and only put the patient to unnecessary pain, hurry the impending fatal catastrophe, and bring discredit upon surgery. I have frequently observed, that in persons of this complexion the usual period of the accession of cancer is anticipated; for instance, I have met with cases of cancer of the uterus in young women of this complexion, from twenty to thirty years of age, and in one case, cancer of both breasts in a child twelve years of age. Two aunts of this girl died of cancer in the breast, and the whole family were remarkable for their anæmic appearance. I have also remarked, that in persons of this complexion assailed by cancer, it always makes an unusually rapid progress, so that I have known instances of women of this

---

* Feb. 5, 1840.

appearance die of the disease in four or five months from the time it was first observed. In these persons, it quickly extended from one breast to the other, also into the axillæ, and from thence onwards to the back underneath the scapulæ. While on the contrary, in some persons of a healthy appearance, the cancerous tumour would remain for years almost stationary, unattached to the skin or parts underneath."

Mr. Carmichael next establishes or endeavours to establish the analogy between parasitical animals and the malignant depositions.

The very same causes, he observes, which only excite the symptoms of scrofula in the human species, seem to be capable of producing tubercles and hydatids, in the lower animals of the mammalia class. Thus animals confined in menageries, deprived of exercise, are constantly found to be infested with hydatids and tubercles in their various organs. Swine are subject to a disease called in them the measles, which is owing to numerous hydatids of the cysto-cercus species, that is, an hydatid, furnished with a neck, head, and suckers, which infest the muscular flesh and cellular membrane, while they have never been found in the animal in its wild state, when in the full enjoyment of exercise. The dairy cows of Paris, deprived of all exercise, and confined incessantly to sheds, are also infested with both tubercles and hydatids. Sheep fed upon low marshy soils, and in very wet seasons, are particularly liable to hydatids in their brains, and a peculiar animal termed by victuallers, flukes, and by zoologists, fasciolæ hepaticæ. But we can produce at our pleasure, both tubercles and hydatids in animals, by depriving them of the genial influence of the solar rays, of that exercise so necessary for the due performance of their various functions—by permitting them only to breathe a damp unwholesome atmosphere, and by feeding them on inappropriate indigestible aliment. Jenner and Baron have produced hydatids and tubercles in the course of a few weeks, in the lungs and livers of rabbits, by subjecting them to this treatment.

" The various facts therefore, just adduced, all prove that the very same causes which only occasion scrofula in the human species, induce the formation of hydatids and other parasites, intermingled with tubercles in the inferior animals; hence, we have reason to infer that man possesses a greater conservative power against the production of parasites than the other mammalia, and we find that the inferior animals are very subject to the visitation of more perfectly formed parasites in their solid tissues than man—for instance, the cysto-cercus hydatid, furnished with a head, neck, and suckers, so common in brutes, has been very seldom found in man, and the fasciola hepatica so common in sheep, has never been found in the human liver. The simple acephalocyste hydatid, a mere membranous bag, containing a clear fluid invested with its proper cyst, and multiplying by the productions of minute hydatids on its interior surface, is the parasite most frequently met with in the solid tissues of the human species.

The independent vitality of tuberculous and carcinomatous masses, although constantly found intermingled with hydatids in the bodies of the lower animals, and sometimes in the human frame, has not, I admit, been proved; but this view of their nature, accounts in so satisfactory a manner, for all the symptoms and phenomena of these malignant productions, which is not afforded by any other hypothesis, that I can not but regard it as a fact, although not at present admitting of demonstrative proof. On this theory alone, we can account for cancer being sometimes a mere local disease, arising from these local causes which injure the organization of a part—while at other times, it is so obviously a constitutional malady, that any attempt at extirpation in general, only increases its ravages; for, though we should be successful in removing it from one place, it will make its appearance in another. On this principle we can well conceive why the breasts and uteri of women, just having passed the period of bearing children, should be particularly liable to the production of those parasitic animal fungi, at a time when the structure of those organs must undergo some diminution of vitality, in consequence of their becoming useless appendages to the female system. Andral observes, ' there are some entozoa that consist of nothing but a parenchymatous mass, without any perceptible organs.' Now, as he does not state what the entozoa are, to which he thus alludes, I presume he could only mean the tuberculous or malignant masses in question. Doctor Farre in his work upon organic diseases of the liver, gives his opinion without hesitation, that a fungous or medullary form of tubercle of that viscus was in possession of a proper vitality, and developed itself by its own innate powers.

Laennec is equally decided respecting the independent vitality of tubercles, and although opposed by Cruveilhier, Majendie, Lombard, Carswell, and others, I am persuaded, that future pathologists will adopt those opinions. Doctor Baron in his work on tuberculated accretions, is obviously another supporter of this doctrine, which I understand, is every day gaining proselytes, but particularly amongst the searching and indefatigable pathologists and naturalists of Germany, so that I feel in no small degree strengthened in a doctrine which I espoused so long ago as 1806, when the first edition of my essay on cancer made its appearance, and in which I had no predecessor but Dr. Adams, who suggested my theory, though in the details I differed widely from him."

Dr. Carmichael combats the argument advanced against him, that tuberculous matter has been found in the blood: he asserts that it has only been discovered in the veins which have absorbed it.

" Tubercles," as I observed at a meeting of the British Association in 1836, " have no connexion by means of vessels with the surrounding tissues in which they are imbedded. This is apparent from the preparations before you; they are commonly found in regular circumscribed masses. They at first, generally before they undergo any transmutation, have the appearance of semi-transparent vesicles; even those that are opaque, on a close examination, I have often found to be hollow; in fact they are thickened vesicles or cysts. But even should they not present the vesicular form, but appear as solid masses without connexion of vessels with the surrounding parts, I do not see why their solidity should be an objection to their possession of an independent vitality. They may continue in this state for months, nay years, without their presence being suspected. As long as they themselves retain life, they do not occasion any stimulus or disturbance in the parts in which they have their nidus, to throw them off; but when they die they act like extraneous bodies (as we know from the facts respecting the guinea-worm) and occasion local inflammation and general disturbance of the system. They soften afterwards, and if situated in the lungs may be expectorated."

He next passes to melanosis, and to *Medullary Fungus.* He particularly insists that, *this medullary fungus is like pulmonary tubercles impervious to injection, and like them first softens in the centre.* From Medullary Fungus he turns to *Scirrhus.* He notices its varying consistence. In making, he says, a section of a cancerous tumour, and pressing it forcibly in your hand, you will find, in almost every instance, that medullary matter will be forced out in distinct spots on the surface of the section thus made, indicating that it is contained and encircled in the firmer cartilaginous substance of the cancerous mass. This foreign production, whether in the first instance medullary or cartilaginous, begins in a point, and extends from thence like radii from a centre; so that, without being contained in a cyst, it penetrates the neighbouring tissues, extending in that direction where it meets with least resistance. Without the advantage of accurate microscopic observation into the structure of this foreign morbid growth at the very commencement, the difficulty of obtaining which is obvious, we can not say, with any degree of certainty, whether the medullary or cartilaginous substance is the first production. But if it be granted that cancer is an animal fungus, possessed of independent vitality, he would conclude that the medullary substance is the primary formation, and the true parasite, as all agree that it does not admit of being injected; but there is much difference of opinion respecting the admission of injection into the more firm cartilaginous substance, which forms intersections of the carcinomatous mass, and it in consequence exhibits an appearance that has been aptly compared to the section of a radish. Mr. Carmichael has often attempted to inject it, but in vain. If, however, we slice with a knife this morbid structure in a living person, an oozing of blood will follow, which would evince that there is in it a minimum degree of circulation of red blood. Slices, he adds, of those malignant masses, taken from living animals before softening or decomposition occurs, and submitted to microscopic observation, might afford considerable information relative to their intimate structure and true nature. If it is found that the medullary substance is the primary formation, not admitting of injection, and that the scirrhous is secondary, possessing the lowest degree of circulation, it may be presumed that the latter substance is formed by the surrounding tissues to insulate this foreign growth, and is therefore analogous to the cysts which contain hydatids in the fasciolæ hepaticæ in sheep and other animals.

These are the principal parts in the Lecture before us which bear on the hypothesis which Mr. Carmichael so ingeniously advocates. They will be read, we think, with interest by those who are not already in possession of his views.

---

### DR. GOLDING BIRD ON THE EMPLOYMENT OF THE ACTIVE PRINCIPLE OF ELATERIUM IN MEDICINE.*

M. Morrus discovered the existence of a white crystallizable matter in elaterium, a formula for preparing which is given by Soubeiran. Dr. Brett informs Dr. Bird that the best mode of preparing elaterine is to boil the crude drug in alcohol, and evaporate the fine green tincture thus obtained to dryness; a mixture of green resin, with a bitter matter soluble in water and elaterine, is left, and on digesting the whole in a solution of potass, the two former substances are dissolved, and the latter is left in the form of a white crystalline powder. Its taste is bitter, it is scarcely soluble in water, it dissolves in boiling alcohol, and, if in excess, is precipitated by cooling (ʒij. of rectified spirit are barely sufficient to hold one grain in solution when cold); it dissolves in ether in small quantities only; in spiritus etheris nitrici it dissolves with the aid of heat. Water precipitates elaterine from its solutions in alcohol and spirits of nitre, unless it is present in very small quantities; in liquor potassæ it does not dissolve, at least in any appreciable quantity, nor does it appear to yield to the solvent power of dilute acids.

From a series of experiments on the most convenient mode of forming a preparation of elaterine for medicinal purposes, so as to permit its administration in minute doses, the following formulæ were ultimately adopted.

*Solutio Elaterinæ.*

℞. Elaterinæ, gr. iv. Spiritus rectificati, f℥iv. Solve ope leni caloris, fʒss. Continet gr. 1-16 Elaterinæ.

*Pulvis Elaterinæ compositus.*

℞. Elaterinæ, gr. iv. Potassæ bitartratis, ʒx. ℈j. Misce accuratissime ℈ss. continet gr. 1-16 Elaterinæ.

After many trials 1-16th of a grain appeared a fair dose to begin with. Given

---

* Med. Gazette, March 13, 1840.

every two hours, two, or at most three or four doses, always proved active, producing copious watery evacuations without griping, or any inconvenience, except in some cases vomiting, where great gastric derangement had previously existed. During the action of the medicine, slight increase in the frequency of the pulse usually occurred, a circumstance generally observed during the administration of Elaterium. Dr. Bird adds; that the elaterine is not liable to the inconstancy of action which characterizes the crude elaterium, and does not produce vomiting or griping.

---

### Case of Circumscribed Aneurysm of the Brachial Artery, after Venæsection, cured by Pressure. By Henry James Johnson.

Perhaps the following case, when taken in connexion with others of a similar character which have been published, may encourage surgeons in the application of pressure to aneurysms of the brachial artery.

A young gentleman, seventeen years of age, was sent to me by Dr. Graham, of Croydon, on the 7th of July, 1838.

There was a circumscribed aneurysm of the brachial artery, just at the point where the vessel dips under the aponeurotic expansion from the tendon of the biceps. The aneurysmal tumor was oval, and about an inch and a quarter in its long diameter—it was partly covered by the aponeurosis. The pulsation was strong—it ceased totally on pressure of the brachial artery above, and returned with rapidity when that pressure was removed. The sac could apparently be emptied, so that there was no evidence of the existence of coagulum in it.

The cicatrix of a puncture in the median basilic vein, just over the aneurysm, was visible; there was no communication between the cavity of the vein and the aneurysm. There was no indication of high bifurcation of the artery.

The young gentleman was delicate, but did not appear to be otherwise out of health.

He had been bled at Braintree for inflammation of the lungs in the early part of May. The blood "spouted far." There was no difficulty in stopping it, but the arm was bound up very tightly. He first discovered the aneurysm a fortnight afterwards, and it had gradually increased since.

The case appeared a favourable one for the trial of pressure, and the patient, as well as Dr. Graham, was anxious for it.

I began with bandaging the fore-arm, from the fingers up to the bend of the arm. Some stripes of soap-strapping were then put round the latter. Over the soap-strapping, a stripe or two of which were turned with the plaister *outwards*, was placed a pad, laid upon the aneurysm, and secured like the pad of a tourniquet, by a slit fillet. Over all, I applied a roller from the hand to some distance up the arm. The effect of these bandages was not only to compress the aneurysmal tumor, but by the confinement of the hand and fore-arm to diminish the quantity of blood going to them, as well as to exert some degree of pressure on the brachial artery itself.

*Low living, rest, and occasional saline aperients, were enjoined.*

I changed the bandage daily until the 13th, when I took off the pad. The aneurysm had decreased in size, and the pulsation both in it and in the brachial artery above it was reduced. The pad, &c. were re-applied, and not renewed until the 18th. I had hitherto debarred the young gentleman from meat, but he now felt so low, that I permitted him to take it every other day.

On the 24th, the aneurysm was not half its former size; it was solid to the touch, and the pulsation in it was scarcely appreciable. The thrill in the brachial artery was remarkably diminished. I now allowed him meat every day. The bandages were continued as before.

It would be useless to make any further diurnal reports. Suffice it to say, that, in the course of another week, pulsation in the aneurysm had totally ceased—that this progressively diminished in size, and in the course of two or three months could not be recognized—that the brachial artery ceased to present any thrill—that the arm regained its usefulness—and, in short, that the success of the treatment was complete.

I regret, however, to add that last year this young gentleman called on me with unequivocal symptoms of hypertrophy and dilatation of the heart. I can scarcely conceive it possible that either the aneurysm or its cure could have operated in the production of the cardiac disease; though possibly the fright and anxiety consequent on the accident might have done so.

8, Suffolk Place, March 20, 1840.

